# Continuous Measurement of Cerebral Oxygenation with Near-Infrared Spectroscopy after Spontaneous Subarachnoid Hemorrhage

**DOI:** 10.5402/2012/907187

**Published:** 2012-11-14

**Authors:** Homajoun Maslehaty, Ulf Krause-Titz, Athanassios K. Petridis, Harald Barth, Hubertus Maximilian Mehdorn

**Affiliations:** Department of Neurosurgery, University Hospitals Schleswig-Holstein, Campus Kiel, Arnold-Heller-Straße 3, 24105 Kiel, Germany

## Abstract

*Objective*. The aim of our prospective study was to investigate the applicability and the diagnostic value of near-infrared spectroscopy (NIRS) in SAH patients using the cerebral oximeter INVOS 5100C. *Methods*. Measurement of cerebral oximetry was done continuously after spontaneous SAH. Decrease of regional oxygen saturation (rSO_2_) was analyzed and interpreted in view of the determined intrinsic and extrinsic factors. Changes of rSO_2_ values were matched with the values of ICP, tipO_2_, and TCD and the results of additional neuroimaging. *Results*. Continuous measurement of rSO_2_ was performed in nine patients with SAH (7 females and 2 males). Mean measurement time was 8.6 days (range 2–12 days). The clinical course was uneventful in 7 patients without occurrence of CVS. In these patients, NIRS measured constant and stable rSO_2_ values without relevant alterations. Special findings are demonstrated in 3 cases. *Conclusion*. Measurement of rSO_2_ with NIRS is a safe, easy to use, noninvasive additional measurement tool for cerebral oxygenation, which is used routinely during vascular and cardiac surgical procedures. NIRS is applicable over a long time period after SAH, especially in alert patients without invasive probes. Our observations were promising, whereby larger studies are needed to answer the open questions.

## 1. Introduction

Delayed cerebral ischemia (DCI) is the major cause of morbidity and mortality in patients suffering from spontaneous subarachnoid hemorrhage (SAH). Besides increasing importance of newer aspects like brain injury, inflammation, and microthrombosis and their influence on DCI, cerebral vasospasm (CVS) remains an important therapeutically target. Therefore, monitoring of cerebral blood flow (CBF) and oxygenation is a substantial and to improving issue in multi-modal intensive care [1–11]. Diverse therapeutically approaches have focussed bedside brain monitoring for early detection of hypoperfusion of the brain. Primarily, it is the measurement of intracerebral pressure (ICP), partial tissue oxygenation (tipO_2_), regional cerebral blood flow using thermal diffusion flowmetry and micro-dialysis—being invasive measurement method. As a noninvasive measurement tool one or two channel EEG adhesive electrodes are used to monitor the sedation depth [2, 11–13]. As another noninvasive but discontinuous bedside measurement method transcranial Doppler (TCD) is also available, with well-known deficits like investigator dependence and weak correlation of elevated blood flow velocity and symptomatic CVS [14, 15].

Near-infrared spectroscopy (NIRS) enables continuous and noninvasive measurement of regional cerebral oxygen saturation (rSO_2_) via absorption of near-infrared light by oxyhemoglobin (HbO), deoxyhemoglobin (Hb), and cytochrome oxidase [1, 16–18]. NIRS is frequently used in cardiac and vascular surgeries and during neuroendovascular procedures as well, achieving good and reliable results, though, its application is confined to the periprocedural and short time of the postprocedural stage [16, 19–21]. Long-term measurement of rSO_2_ for early detection of hypoperfusion of the brain after SAH is not yet widespread, so that the aim of our prospective study was to examine the applicability and the diagnostic value of NIRS used continuously in SAH patients over the estimated CVS period.

## 2. Methods

### 2.1. INVOS-System

**Figure 1:**
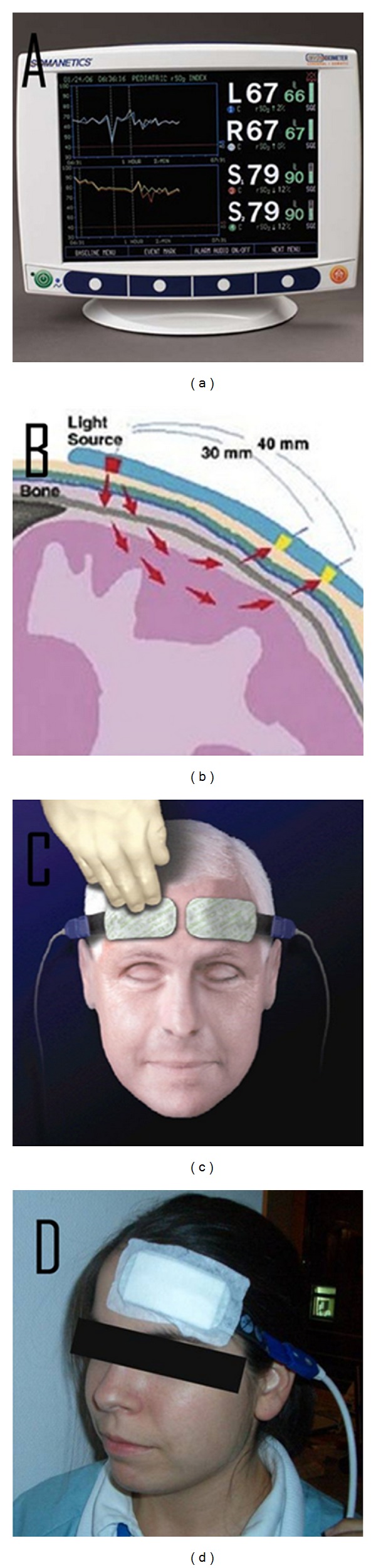
(a) INVOS monitor; (b) schematic description of working process; (c) and (d) application oximetry sensors to the forehead.

In this study we used the INVOS 5100C oximeter (Somanetics), which provides a continuous, noninvasive and real time measurement of cerebral oxygenation (Figure 1(a)).

The near-infrared wavelengths are generated by a light source of the sensor and penetrate the skin and the bone. Within the brain tissue in 3 cm depth the light is either absorbed or reflected to the two detectors of the sensor (Figure 1(b)). Since hemoglobin within the detection field consists of venous blood in about 75%, arterial blood in about 20%, and capillary blood in about 5%, clinical interpretation of the values can be considered as a venous measurement. Red dyed hemoglobin (HbO) displays the highest absorption of used wavelengths. Hence, the red shade of each hemoglobin molecule shows the containing oxygen concentration. The portion of reflected data gives the relative concentration of deoxyhemoglobin and the overall level of hemoglobin, from which the regional oxygen saturation (% rSO_2_) is calculated. The continuous measured data are displayed on the monitor with an update every five seconds.

### 2.2. Study Protocol

This study was approved by the Ethics Committee of the University of Schleswig-Holstein, Campus Kiel. 

We included patients with spontaneous SAH, diagnosed by CT scanning or analysis of cerebrospinal fluid via lumbar puncture within 24 h after occurrence of symptoms in our study. After diagnosis, digital subtraction angiography (DSA) or CT angiography was performed for detecting the bleeding source. After treatment of the bleeding source (clip occlusion or coil embolization), all patients were treated in the neurosurgical ICU with continuous measurement of the arterial blood pressure, pulse rate, oxygen saturation, and continuous recording of electrocardiogram. The neurological condition in the continuing course was assessed by the physician at the ICU closely, using the Hunt and Hess scale (H&H). Appearance of blood on CT was scored using the Fisher classification. 

TCD was performed daily to measure the flow velocity of the intracranial arteries. CVS was determined as acceleration of blood flow velocity > 120 cm/second. Sedated and intubated patients with mechanical ventilation received intracranial probes for continuous measurement of ICP and tipO_2_ additionally. External ventricular drainage was done in cases of occlusive hydrocephalus or hydrocephalus like ventricular system. In cases of elevated ICP due to insufficient sedation, one-channel EEG electrodes (BIS) were applied additionally to measure the sedation depth. 

After admission of the patients in the ICU, the forehead of the patients was cleaned up with alcohol pads, and self-adhesive oximetry strips were applied bilaterally to measure the baseline value of rSO_2_ in 3 cm depth (Figure 1(c)). After setting the baseline value, we started the continuous measurement of rSO_2_ over the estimated CVS period up to the 12th day after onset. Measured data were saved in the memory device of INVOS 5100C and additionally in a memory stick. 

In cases of decrease of rSO_2_ with an obvious cause—for example, removing the strips for personal hygiene, dysfunction of the strips, or removing for neuroimaging—an event mark button was pressed to illustrate this event and to allow correct correlation during data analysis.

The oximetry strips could be removed during nursing activities and were reapplied at the same position. Once the self-adhesive behaviour of the strips diminished, fixation was done by a regular wound plaster to obtain further measurement (Figure 1(d)).

### 2.3. Threshold Values and Data Interpretation

Desaturation below 50% or decrease of rSO_2_ by 20% from the baseline value was estimated to be critical; rSO_2_ below 40% or decrease by 25% was assumed to be associated with DCI and neurological deficits.

For correct analysis of the measured values, intrinsic and extrinsic factors were considered exactly. Intrinsic factors were determined to be mean arterial blood pressure (MAP), hemoglobin (Hb), peripheral oxygen saturation (SaO_2_), partial carbon dioxide pressure (pCO_2_), and core temperature (*t*) [22]. Extrinsic factors were head positioning, correct position and adhesion of the strips, and correct connection of the strips to the INVOS device. In case of decrease of rSO_2_ intrinsic and extrinsic factors were controlled and equalized, before terming the rSO_2_ value pathologic and performing further diagnostic evaluations. 

For analysis, the recorded data were transferred and represented graphically in the Microsoft Office Excel program. Furthermore, changes of rSO_2_ values were matched with the values of ICP, tipO_2_, and TCD and the results of additional neuroimaging. 

## 3. Results

Continuous measurement of rSO_2_ was performed in nine patients (7 females, 2 males). DSA revealed an aneurysm as the source of hemorrhage in 7 patients (three aneurysms of the anterior communicating artery (ACoA), one middle cerebral artery (MCA) aneurysm, one aneurysm of the posterior cerebral artery (PCA), and one aneurysm of the pericallosal artery). The bleeding source was unclear in one case after DSA (SAH of unknown origin). In the last case DSA was not performed due to poor clinical condition with cessation of CBF diagnosed by perfusion-weighted CT scanning (PW-CT) and brain death two days after admission. 

Five aneurysms were treated with clip occlusion, and two aneurysms were embolized with coils. The distribution to the H&H and Fischer (F) scale was as follows: H&H1:*n* = 1, H&H2: *n* = 3, H&H3:*n* = 0, H&H4:*n* = 3, H&H5:*n* = 2; F1:*n* = 1, F2:*n* = 0, F3:*n* = 3, F4:*n* = 5. 

Mean measurement time of rSO_2_ was 8.6 days (range 2–12 days). The clinical course was uneventful in 7 patients without occurrence of CVS and ischemic strokes. In these patients, NIRS measured constant and stable rSO_2_ values without any significant alterations. 

Special findings and characteristics of NIRS application are illustrated in three displayed cases.

**Case 1**

**Figure 2:**
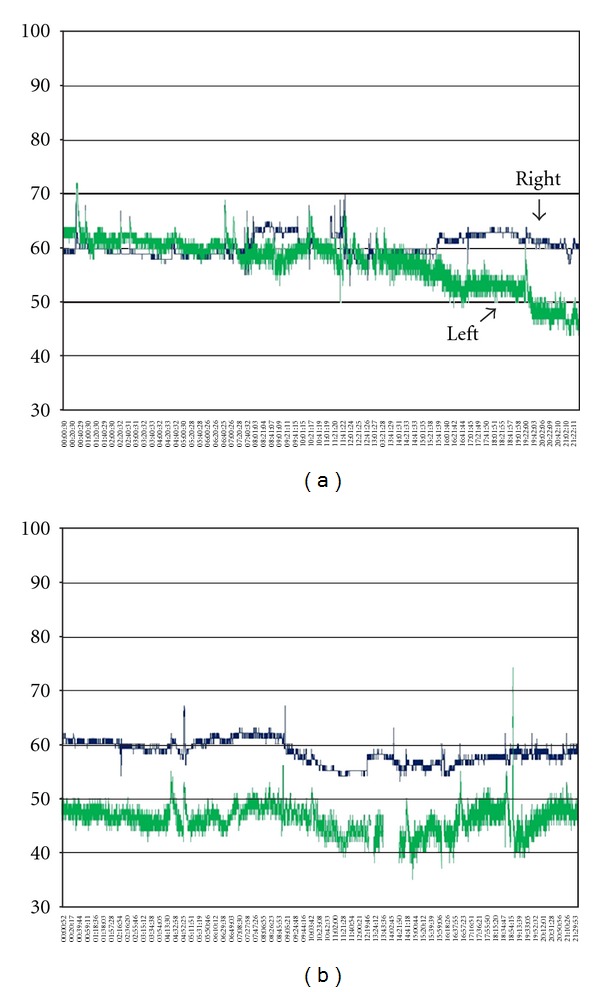
NIRS shows progressive left-sided decrease of rSO_2_.

**Figure 3:**
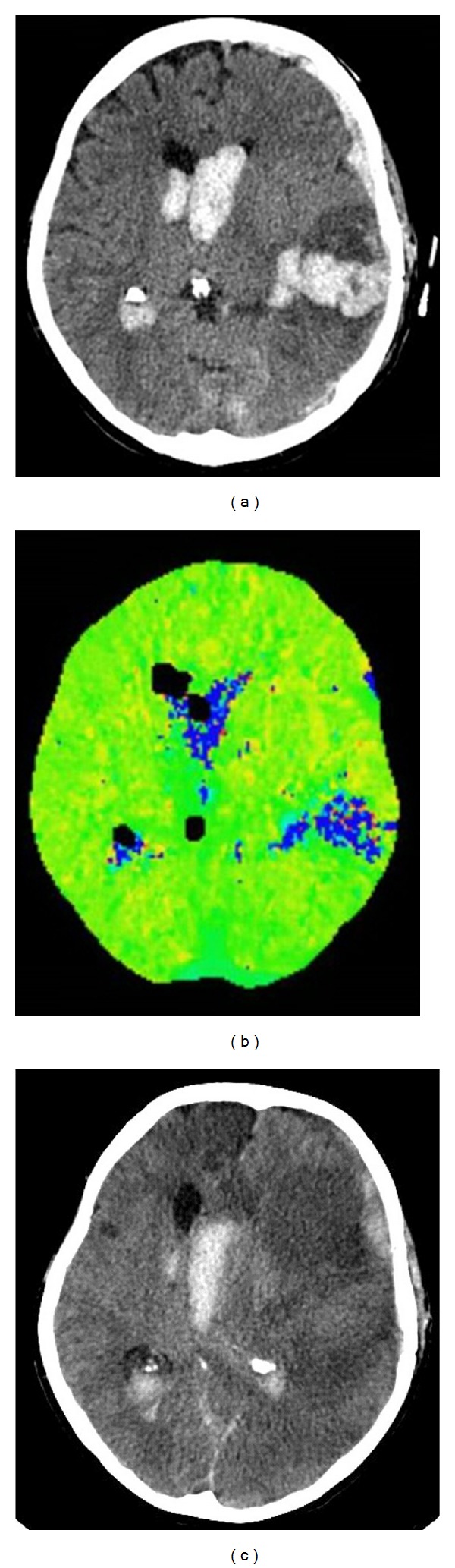
(a) and (b) native CT and perfusion-weighted CT scan at time of left-sided desaturation on day 5 without new ischemic stroke; (c) native CT scan four days later showing distinct left hemispheric ischemic stroke with midline shift.

A 70-year-old female patient presented with SAH H&H grade 5, Fisher grade 4 due to a ruptured left-sided PCA aneurysm with intracerebral and intraventricular hemorrhage. The aneurysm was treated surgically, and the patient remained sedated and intubated, receiving mechanical ventilation postoperatively. In the continuing course, TCD showed elevated blood flow velocities of both MCA and ACA arteries of up to 200 cm/second. Despite triple H therapy and nifedipine application, NIRS showed left-sided decrease of rSO_2_ below 40% on day 5 after onset (Figure 2). Intrinsic and extrinsic factors were normal at that time (MAP 127 mmHg, SaO_2_ 99%, FiO_2_ 60%, *t* 38.3°C, pCO_2_ 35%, and Hb 11.3 g/dL). Left frontal applied ICP probe showed no significant changes at the same time (ICP 11 mmHg, CPP 118 mmHg). Subsequently performed native CT and PW-CT scans showed neither perfusion deficits nor ischemic stroke (Figures 3(a) and 3(b)).

Left-sided rSO_2_ values remained on a low level with further decrease. Two days later, ICP increased slowly and reached the maximum of 39 mmHg on day twelve after onset. In parallel to this right-sided rSO_2_, values decreased as well. Newly performed CT scan showed a marked left hemispheric ischemic stroke with shift of the midline strictures and signs of brain herniation (Figure 3(c)). In consideration of the poor clinical condition, the age, and occurrence of distinct ischemic stroke, we decided to limit the therapy. The patient died on day twelve after onset.

**Case 2**

**Figure 4:**
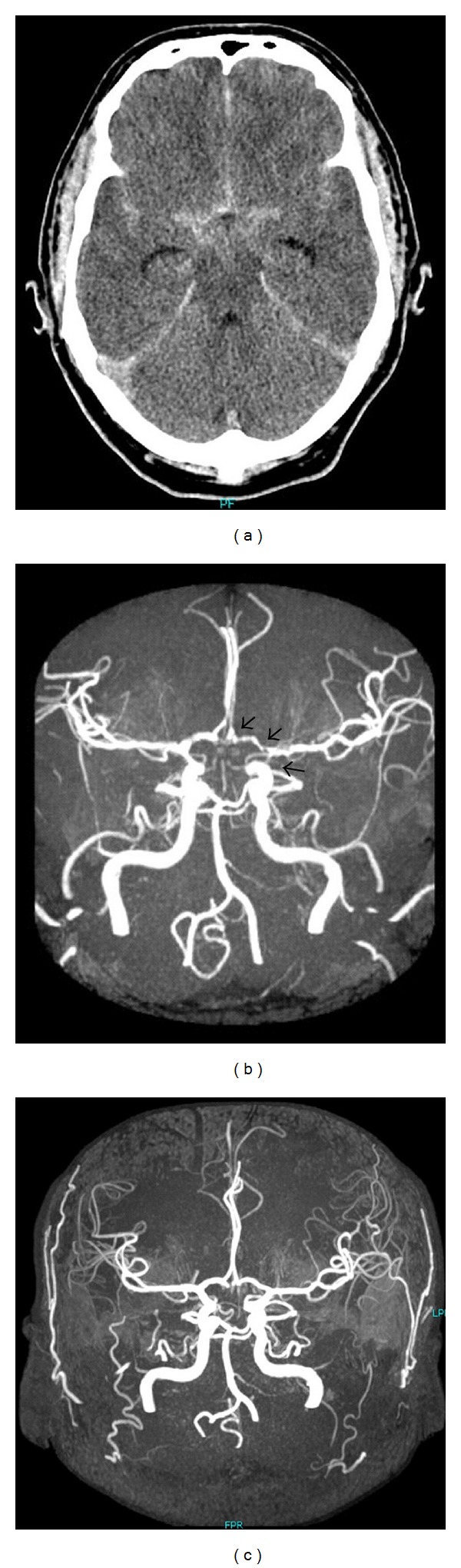
(a) initial CT scan shows SAH; (b) MRA shows radiologic CVS of left-sided ACA and ICA (arrows); (c) 6-month followup MRA shows normalized vascular patterns.

**Figure 5:**
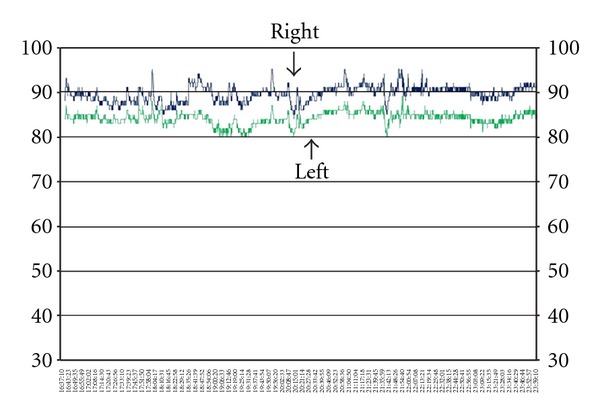
NIRS shows stable and normal ranged rSO_2_ values.

A 42-year-old male patient presented with SAH H&H grade 2 and Fisher grade 3 due to a ruptured aneurysm of the ACoA (Figure 4(a)). The aneurysm was treated via coil embolization. In the continuing course, the patient suffered from headaches, but he was alert without neurological deficits at all times. NIRS showed normal and stable rSO_2_ values (Figure 5). In the continuing course, TCD showed elevated blood flow velocities of the left ACA and MCA up to 220 cm/second. Although performed magnetic resonance angiography (MRA) showed radiological spasm of the left ICA and ACA (Figure 4(b)), the clinical condition of the patient remained stable without deterioration. Further on, NIRS showed stable rSO_2_ values without significant desaturation. In line with performed mild triple-H therapy and oral nifedipine application, TCD values normalized in the continuing course, and the patient could be discharged without any neurological complaints. At the six months follow-up examination, the patient was still in a good condition, and he was working again. MRA showed normalized vascular patterns (Figure 4(c)).

**Case 3**

**Figure 6:**
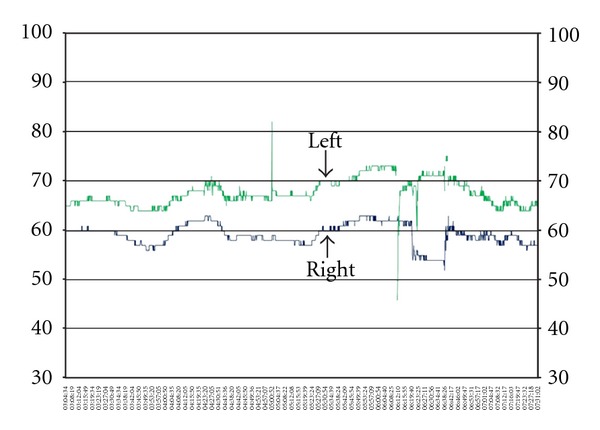
Paradox measurement of relative stable and normal rSO_2_ values in a brain dead patient.

In this case, it was a 14-year-old girl, who was found comatose at home with dilated and nonresponsive pupils. CT scan showed massive SAH with huge cerebral edema. PW-CT showed cessation of CBF, and TCD showed abnormal reverberating flow, indicating distinct increased ICP. NIRS strips were applied for two days and measured interestingly rSO_2_ values of 63%. Over the measurement period of about 48 hours rSO_2_ values were very stable within the range of 60–70%, (Figure 6) without common fluctuations as seen in other patients. Two days after onset the patient was pronounced brain dead.

## 4. Discussion

Application of NIRS is done routinely during vascular and cardiac surgical procedures. The results of several studies have shown that monitoring of rSO_2_ decreases the risk of procedure-related cerebral desaturation and improves the outcome in those patient groups [23–27].

However, continuous measurement of cerebral oxygenation with NIRS after spontaneous SAH is not sufficiently investigated. Just a few studies focused on the application of NIRS after SAH [1, 17, 18]. While Mutoh, Yokose, and Zweifel used the NIRO 200 monitor (Hamamatsu) in their studies, comparable studies with the INVOS system (Sommanetics) are to the best of our knowledge not available. The mentioned studies draw the conclusions that application of NIRS can provide continuous and long-term measurement of rSO_2_ and can give relevant information about the cerebral autoregulation and the effectiveness of performed therapy on CVS.

Concluding the results of our study, we can postulate that measurement of cerebral oxygenation with NIRS is a save and easy to use noninvasive additional measurement tool, which is applicable for long-term measurements in SAH patients. 

It seems to be useful especially in alert patients, in whom invasive probes are not usually used. In general, performance of rSO_2_ measurement was done without major problems in our study, whereby the nursing personnel needed repeatedly well instruction to obtain a smooth functioning process. 

Although our examined patient group is relatively small, the displayed cases show in an illustrative manner the diagnostic relevance of measured values. 

In Case 1, NIRS detected early relevant hypoperfusion of the ACA territory way ahead of increased ICP. Also PW-CT failed to detect perfusion deficits at the same time. The interpretation of measured elevated blood flow velocities by TCD is controversial concerning radiological and symptomatic CVS, as already discussed in other studies [14, 15]. This case illustrated pointedly the diagnostic value of NIRS for early detection of DCI in a sedated and intubated patient.

Vice versa, rSO_2_ values remained consistently stable in Case 2 with proven CVS by TCD and MRA. The patient developed no neurological impairments, so that CVS could be termed radiological. In this case, NIRS was a very important additional measurement tool to control the gathered findings in view of DCI in an alert patient. Even though the neurological examination by a physician probably remains the easiest and most reliable procedure to detect CVS related deteriorations in alert patients, early illustration of changes in cerebral oxygenation before clinical manifestation is desirable and might be provided by NIRS.

Case 3 can be discussed controversially. We measured rSO_2_ values above 60% in a brain dead patient. Similar observations were made by Kyttä and coworkers [28]. The investigators examined six brain dead patients with NIRS and measured relatively normal rSO_2_ values and brain desaturation dependent on the level of mechanical ventilation. Hence, the authors suggested an additional extracranial contribution of measured rSO_2_ values and restricted the diagnostic relevance of NIRS in the diagnostic protocol of brain death [2]. Although new generations of NIRS devices claimed to exclude the extracranial component effectively, and the tissue oxygenation index was shown to be independent of hemoglobin concentration, skull thickness, and the area of the cerebrospinal fluid layer underlying the sensors [18, 29], our findings remain poorly explained, raising further questions in this area of research. A possible explanation could be that NIRS measured the remaining desoxgenated haemoglobin in the brain, which does not underly the blood circulation. Thus, this attempted explanation could state the relative constant rSO_2_ values without alterations. According to this particular case, it is arguable that application of NIRS is unsuitable in brain dead patients. 

Different aspects should be considered, which could lead to limitation of its applicability and interpretation. First of all, it is to note that the measurement of cerebral oxygenation mainly involves the ACA territory. The important MCA territory is only partly captured, if the strips are applied to the forehead. It remains to prove if application of the strips to the temples after head shaving can provide reliable rSO_2_ values of the MCA territory. 

Another problem is the restricted applicability due to limited space on the forehead if the sedation depth has to be measured with EEG electrodes in patients with increased ICP. A solution for this problem could be the combination of NIRS strips and EEG electrodes, which is an object of development.

Fever and sweat of the skin have an influence on the measurement. This could be a problem for continuous and long-term measurement, since infections and fever occur relatively frequently in sedated and intubated patients at the ICU. Hence, measured data could be unsuitable for decision making towards further imaging and therapy. 

Furthermore, it is to note that application of NIRS strips requires a compliant patient. Measurement and data interpretation of an agitated patient is nearly impossible, if the patient dislocates and tears the strips off repeatedly. 

Finally, there is a lack of common consensus about the threshold values in view of necessity of intervention [2, 22, 30, 31]. However, it has already been postulated and is also our opinion that NIRS is suitable for recording trends. Our selected threshold values were based on the information of the manufacturer, which is the result of a large number of other studies. 

It is desirable to have a noninvasive and continuous measurement method of cerebral oxygenation with reliable values. Despite the promising course of our study, further large prospective studies are required to answer the open questions and to test our observations.

## 5. Conclusion

Measurement of rSO_2_ with NIRS is a safe, easy to use, noninvasive additional measurement tool for cerebral oxygenation, which is used routinely during vascular and cardiac surgical procedures. NIRS is applicable over a long-time period after SAH, especially in alert patients without invasive probes. Extrinsic and intrinsic factors have to be considered exactly to enable correct data interpretation. Under consideration of our relative small patient group, the measured values seem to be relevant and reliable. However, further large studies are needed to test our observations and to obtain recommendations for its application in SAH patients.
